# Role of Psychosomatic Medicine in Complex Medical Cases: A Case Study of a Patient With Breast Cancer Who Refused Mastectomy

**DOI:** 10.7759/cureus.61343

**Published:** 2024-05-30

**Authors:** Meaad F Alatawi, Abdulaziz Al-Saif, Fahad D Alosaimi

**Affiliations:** 1 Department of Psychiatry, College of Medicine, King Saud University, Riyadh, SAU; 2 Department of General Surgery, College of Medicine, King Saud University, Riyadh, SAU

**Keywords:** consultation-liaison psychiatry, inflammatory breast disease, breast and endocrine surgery, endocrine oncology, musculoskeletal oncology, bio-psycho-social model, clinical psychiatry

## Abstract

Psychosomatic medicine has been known to play a pivotal role in the management of complex medical cases by providing a bridge between the physical disease and psychological distress. This case study focuses on a 42-year-old Saudi female diagnosed with breast cancer and generalized anxiety disorder. In addition to anxiety, the patient had a history of obsessive-compulsive personality traits, which contributed to her reluctance to undergo mastectomy. Significant challenges and noncompliance with treatment were caused by her unwillingness and inadequate interaction with the medical team. An integrated multidisciplinary strategy including psycho-oncological interventions was necessary because of the complexity of this case. Communication issues were addressed through the concerted efforts of the specialist teams. A comprehensive patient-practitioner understanding was established, which enabled the teams to persuade the patient to undergo surgical intervention. Overcoming her initial resistance, the patient eventually complied with the treatment plan, leading to a successful surgery. Postsurgical evaluations using the Hamilton Anxiety Scale indicated a significant reduction in anxiety levels. This case underscores the critical contribution of psychosomatic medicine to healthcare, especially in challenging situations that demand additional resource allocation, further highlighting the importance of an interdisciplinary approach, efficient communication, and patient-practitioner rapport in healthcare outcomes

## Introduction

Psychosomatic medicine (consultation-liaison psychiatry) explores the intricate connection between physical health and psychological well-being and plays a significant role in complex medical cases [1]. The study and application of comprehending how psychological variables might affect physical health and vice versa are addressed in this field. It involves a comprehensive assessment and treatment of individuals, considering the interplay between their emotional, cognitive, and physiological states [2]. The influence of psychosomatic medicine becomes particularly pronounced when a comprehensive patient-centered approach is necessary to bridge the gap between physiological and psychological health [2]. Within the realm of psychosomatic medicine, the area of interest spans a wide spectrum of complex medical conditions, with particular emphasis on cases displaying close interaction between the mind and body, exploring how psychological factors such as stress, anxiety, and emotional well-being can influence the onset, progression, and management of various physical illnesses [3]. A notable subspecialty within this field is psycho-oncology, which focuses specifically on the psychological aspects of cancer care [4]. The background of psycho-oncology varies across countries, with North America and Europe contributing uniquely to the field’s development. In Canada, psycho-oncology gained recognition and prominence in the mid-20th century, evolving into a multidisciplinary approach involving psychologists, psychiatrists, oncologists, and others [3].

The significance of psychosomatic medicine in complex medical cases remains underestimated. Its influence emerges predominantly when a holistic, patient-centered approach is necessary to bridge the gap between physiological and psychological healing [5]. The intricate interconnection between physical illnesses and psychological distress often complicates patient management, leading to interruptions in treatment adherence [6]. This interdependence is particularly obvious in the field of oncology, where patients’ psychological well-being significantly influences their ability to cope with the illness, their reaction to treatment, and, ultimately, the treatment outcomes. The integration of psycho-oncological interventions is fundamental for effective patient care in such cases [7].

Breast cancer is the most commonly diagnosed malignancy among women worldwide and the leading cause of cancer-related death [8]. A systematic review of the epidemiology of cancer in Saudi Arabia from 2010 to 2019 confirmed that breast cancer is the most common cancer in Saudi Arabia as well [9]. A local community study found that there was suboptimal knowledge and practice related to breast cancer in Saudi Arabia [10]. Furthermore, another local study found that higher distress among cancer patients is associated with their decision-making [11]. Cultural beliefs and attitudes toward cancer, treatment options, and mental health may also play a significant role in the decision-making process. In conservative cultures like Saudi Arabia, family and community roles are often highly valued, and the patient may feel a strong sense of duty and responsibility toward her family and community. Traditional gender roles and expectations may influence her perception of femininity and body image, potentially impacting her decision to refuse any disfiguring interventions such as mastectomy [11].

Recent clinical guidelines recommend that patients be given information on diagnosis and treatment choice repeatedly (both verbally and in writing) in a comprehensive and easily understandable manner using reliable, patient-centered websites as well as involving patients actively in all therapy-management decision-making [12].

This case study explores these dimensions in the context of a 42-year-old Saudi woman diagnosed with breast cancer. The challenges faced in her treatment journey reflected a confluence of psychological factors, the individual’s personal resistance to treatment, and the interpersonal dynamics involving the patient, her family, and the medical team. The clinical picture of surgery was further confounded by the patient’s obsessive-compulsive personality characteristics and elevated anxiety levels. The main objective of this paper is to describe the important role of psychosomatic medicine in the assessment and management of complex medical cases specifically on the treatment decision-making process and overall well-being of patients with breast cancer who refuse mastectomy.

In this report, we describe the intricacies that emerged in this case, emphasizing the importance of effective communication and efficient interdisciplinary teamwork as well as the critical role that psychosomatic medicine plays in managing cases like this one. This narrative underlines the necessity of recognizing and addressing comorbid psychological distress in patients with severe physical illnesses to ensure effective patient care and treatment outcomes [13].

## Case presentation

A 42-year-old married Saudi woman, a mother of three kids, a teacher, and considered a pillar of her family and community, was profoundly affected by the diagnosis of breast cancer eight months before her presentation in the psycho-oncology clinic. As a teacher, she had a stable income but was likely not financially independent from her spouse or family.

Her husband played a significant role in her life as a supporter. Her recent suffering started in 2022 when pathological assessments of her right breast revealed stage 1 invasive ductal carcinoma with tubular features. After receiving this diagnosis, her health, the possible ramifications on her personal and professional life, and the welfare of her children became significant concerns for the patient. Her heightened anxiety was clinically significant, with a score of 9 on a 10-point anxiety scale, and was evident to her healthcare providers and family, thereby necessitating psychosomatic intervention which was unfortunately delayed for eight months. The intervention included primarily a comprehensive psychosomatic assessment, including the evaluation of current functional impairment, the current psychosocial situation, and the coping mechanisms utilized by the patient. Empathy and full understanding of psychological response to medical illness were important aspects of the intervention [14].

Upon assessment, her past psychiatric history revealed a diagnosis of adjustment disorder and obsessive-compulsive personality traits 10 years before her current presentation. She had adhered to an unknown psychotropic medication regimen and cognitive behavioral therapy for a brief period of eight weeks, discontinuing both prematurely due to side effects and scheduling difficulties. Additional complexities in her medical history included dyslipidemia and early-onset menopause.

Her breast cancer diagnosis in 2022 initiated a potent cycle of denial and resistance to treatment, a complex mix of obsessive-compulsive traits, heightened anxiety, and mistrust toward the healthcare team. Symptoms included excessive rumination, concerns about her health and children, irritability, bodily discomfort, restlessness, and a fear of mortality. Notably, she did not exhibit signs of depression, suicidal thoughts, homicidal tendencies, or psychosis, but encountered difficulties in decision-making regarding her medical situation. The patient’s resistance was exacerbated by ineffective communication between both the patient and her husband with the healthcare providers, leading to missed appointments and delayed treatments for eight months. Miscommunication challenges were exacerbated by the husband of the patient, who displayed inappropriate behavior during the initial stage of communicating the cancer diagnosis and discussing the treatment options. His actions toward the Attending Fellow and the Primary Consultant were evident as he continuously interrupted and pushed for a delay in the surgery against the patient’s wishes. During this eight-month period of missed appointments and delayed treatments, the patient was staying home and dysfunctional overall. She was not taking care of her family as she used to, not going to work, and less socializing with significant others. Instead, she spent her time googling alternative cancer treatments worldwide and spent thousands of Saudi Riyals on some herbal medicines with no benefits. Then, after strong persistent family pressure, the patient agreed to pursue professional help again. The patient’s niece, who was a medical student, approached the psychosomatic medicine psychiatrist to arrange an urgent appointment for her aunt in his psycho-oncology clinic.

To address the complexities of this case, the psychosomatic medicine psychiatrist not only worked directly with the patient but also maintained continuous communication with the surgical and oncology teams to ensure a unified approach. Following comprehensive assessments, the patient was diagnosed with generalized anxiety disorder (GAD). This was a pivotal finding, emphasizing that the anxiety associated with her obsessive-compulsive traits escalated with the recent cancer diagnosis and was a significant obstacle to treatment adherence. A multifaceted treatment strategy was subsequently devised, which the patient followed for 1.5 months. An important component of the psychosomatic interventions was psychoeducation to deepen the patient’s understanding of her health conditions.

The patient was started on escitalopram 10 mg and lorazepam 1 mg twice daily, with weekly monitoring. The treatment strategy incorporated consultations with psycho-oncology specialists, supportive psychotherapy, and a gradual adjustment of escitalopram and lorazepam dosages to alleviate her symptoms. Furthermore, the patient received psychoeducation, reassurance, and collaborative care with the medical oncology and surgical teams to address her emotional needs and provide comprehensive support throughout her treatment journey. Moreover, supportive psychotherapy of six sessions was conducted by the psychiatry resident trainee under supervision to provide emotional sustenance, enhance coping strategies, and train the patient on relaxation techniques. Medication doses were adjusted to alleviate her symptoms and lorazepam was increased to 2 mg twice daily for two weeks to manage her intense acute anxiety in addition to a long course of escitalopram 20 mg once daily for GAD. Post-treatment, her anxiety level notably decreased to 4 on the 10-point scale.

The psycho-oncology intervention was instrumental in facilitating a trusting environment for the patient and in coordinating closely with the surgical and oncology teams to enhance communication and care. This direct interaction helped not only in mitigating her earlier resistance but also in fortifying the multidisciplinary treatment strategy. Furthermore, the surgeon spent good time with the patient and her husband to provide sufficient education on cancer-related diagnoses and management issues.

As a result of these collaborative efforts, the patient’s hesitancy toward the right mastectomy procedure gradually decreased. Postsurgical assessments using the Hamilton Anxiety Scale indicated a significant reduction in the patient’s anxiety levels from 25 to 9 points, and after a six-month follow-up, the score dropped from 9 to 2 points. Moreover, fortunately, despite delayed surgery, no distant metastasis was discovered. This episode serves as an example of the benefits of psychosomatic medicine in addressing both the physical and psychological elements of the illness, thereby improving patient care and treatment outcomes.

## Discussion

In the case reported here, the Canadian Medical Education Directions for Specialists’ (CanMEDS) roles of communicators, collaborators, and health advocates were vital in addressing the challenges faced [15]. The healthcare team invested extra effort in persuading the patient to take the initial steps toward treatment. Recognizing her anxiety and resistance to surgery, the team worked diligently to build trust, provide reassurance, and alleviate her fears. This advocacy role ultimately secured the patient’s consent for surgery, emphasizing the importance of patient-centered care.

The specialized expertise of psychosomatic medicine specialists within the healthcare team is crucial. This specialist usually demonstrates extra proficiency in the CanMEDS roles of communicator, collaborator, and advocate to address the unique challenges presented by such complex medical cases. Psychosomatic medicine serves as a bridge between the physical and psychological aspects of illness, highlighting the need for specialized interventions beyond traditional referral methods that complex medical cases often require. Complex medical cases are those representing challenges that require a disproportionate amount of effort and attention. They may constitute about 20% of patients compared to 80% of patients who follow straightforward treatment paths, according to the Pareto principle (80/20 rule) [16].

Depression and anxiety can significantly impact a patient’s healthcare journey, with both direct and indirect effects that underscore the importance of their management [17]. They can directly lead to treatment noncompliance, reduced motivation, and energy as well as a sense of hopelessness. In this case, the patient’s initial resistance to surgery could be partially attributed to heightened anxiety levels and a fear of unknown causes of her cancer. Thus, addressing these psychiatric comorbidities is paramount in ensuring treatment adherence and efficacy [17]. The indirect effects of depression and anxiety were equally noteworthy [17].

These psychiatric comorbidities often hinder effective communication between patients and healthcare providers. Patients experiencing anxiety may struggle to articulate their concerns and needs clearly, whereas those experiencing depression may withdraw from active participation in their care. This communication barrier can impede accurate diagnosis and appropriate treatment planning [17]. In a case-control study conducted in Geneva to compare the characteristics of women who accepted surgery versus those who declined, it was observed that 23% declined due to psychological issues [18].

There is a huge variation in individuals’ responses to environmental stressors, for example receiving a diagnosis of cancer. Under stressful situations, the characteristic means of adaptation are heightened [19]. Thus, facing the stress of a medical illness and hospitalization, an obsessional patient, like our patient, could present as overly rigid or controlling. Armed with an understanding of the patient’s personality style, the psychosomatic psychiatrist will be able to reduce the patient’s distress and augment a sense of control.

Importantly, a psychosomatic medicine specialist, in this case, addressed the patient’s psychological distress along with her medical condition, following a holistic approach that integrates psychological support, psychoeducation, and pharmacological interventions, if necessary. This can alleviate the burden of these comorbidities and improve patients’ overall well-being and engagement in the healthcare journey. Effective management not only addresses the direct and indirect effects but also enhances collaboration and communication among healthcare professionals, the patient, and their support system, ultimately leading to more favorable outcomes [20].

However, despite the benefits of this approach, there may be limitations and challenges in case management. Access to specialized psychosomatic medicine services could be limited, and the availability of trained specialists in psychosomatic medicine may be a challenge, leading to difficulties in providing comprehensive care to all patients in need. Additionally, patients may face stigma or cultural beliefs that hinder their acceptance of psychological support, potentially impacting the effectiveness of such interventions. Ensuring patient adherence to psychosomatic interventions, including medication regimens and therapy sessions, can be challenging and may require additional support and monitoring. Coordination among healthcare professionals from different disciplines may present challenges in ensuring seamless communication and integration of care plans.

To address these limitations and enhance case management, potential areas for improvement may include increased training and awareness for healthcare professionals on the importance of psychosomatic medicine and integrated care. Tailoring interventions to align with patients’ cultural beliefs and values can enhance the acceptance and effectiveness of psychosomatic interventions. Empowering patients with knowledge about the benefits of psychosomatic interventions and involving them in decision-making processes can improve engagement and adherence to treatment plans. Additionally, implementing strategies to improve communication and collaboration among healthcare providers, such as regular interdisciplinary team meetings and shared electronic health records, can facilitate coordinated care delivery.

## Conclusions

This case report underscores the critical roles played by communicators, collaborators, and advocates in healthcare, particularly in cases involving intricate medical and psychological components, and how they are indispensable elements in achieving positive outcomes for patients with challenging medical conditions. This case report also emphasizes the expertise of psychosomatic medicine specialists in navigating the intricacies of such complex cases and the significance of a biopsychosocial approach in delivering comprehensive patient care, particularly in areas such as oncology, where psychological burdens can profoundly impact patient outcomes.
